# Antifungal activity of eumelanin-inspired indoylenepheyleneethynylene against *Cryptococcus neoformans*

**DOI:** 10.3389/fmicb.2023.1339303

**Published:** 2024-01-16

**Authors:** Brittney N. Conn, Jacob A. Lieberman, Priscilla Chatman, Kaitlyn Cotton, Martha A. Essandoh, Mohammad Ebqa’ai, Toby L. Nelson, Karen L. Wozniak

**Affiliations:** ^1^Department of Microbiology and Molecular Genetics, Oklahoma State University, Stillwater, OK, United States; ^2^Department of Chemistry, Oklahoma State University, Stillwater, OK, United States

**Keywords:** *Cryptococcus neoformans*, antifungal, novel antifungal compound, EIPE-1, eumelanin

## Abstract

*Cryptococcus neoformans* is an opportunistic fungal pathogen that causes meningitis in >152,000 immunocompromised individuals annually, leading to 112,000 yearly deaths. The four classes of existing antifungal agents target plasma membrane sterols (ergosterol), nucleic acid synthesis, and cell wall synthesis. Existing drugs are not highly effective against *Cryptococcus*, and antifungal drug resistance is an increasing problem. A novel antimicrobial compound, a eumelanin-inspired indoylenepheyleneethynylene, EIPE-1, was synthesized and has antimicrobial activity against Gram-positive bacteria, including methicillin-resistant *Staphylococcus aureus* (MSRA), but not towards Gram-negative organisms. Based on EIPE-1’s antibacterial activity, we hypothesized that EIPE-1 could have antifungal activity. For these studies, we tested EIPE-1 against *C. neoformans* strain H99 and 6 additional cryptococcal clinical isolates. We examined antifungal activity, cytotoxicity, effects on fungal gene expression, and mechanism of action of EIPE-1. Results showed that EIPE-1 has fungicidal effects on seven cryptococcal strains with MICs ranging from 1.56 to 3.125 μg/mL depending on the strain, and it is non-toxic to mammalian cells. We conducted scanning and transmission electron microscopy on the exposed cells to examine structural changes to the organism following EIPE-1 treatment. Cells exposed displayed structural changes to their cell wall and membranes, with internal contents leaking out of the cells. To understand the effect of EIPE-1 on fungal gene expression, RNA sequencing was conducted. Results showed that EIPE-1 affects several processes involved stress response, ergosterol biosynthesis, capsule biosynthesis, and cell wall attachment and remodeling. Therefore, our studies demonstrate that EIPE-1 has antifungal activity against *C. neoformans,* which affects both cellular structure and gene expression of multiple fungal pathways involved in cell membrane stability and viability.

## Introduction

*Cryptococcus neoformans* is an encapsulated fungal pathogen, transmitted frequently via inhalation of environmental spores found in soil, pigeon excrement, and decomposing wood (Levitz, 1991; Kwon-Chung et al., 2014). Infection by the pathogen can result in cryptococcosis, which manifests initially as a pulmonary disease but can also disseminate to the central nervous system (CNS) leading to cryptococcal meningitis (Chang et al., 2004; Shi et al., 2010; Kwon-Chung et al., 2014). HIV patients are primarily at risk of the development of cryptococcosis. They make up 95% of all cases reported in low-to-middle income countries, such as those in Sub-Saharan Africa, and 80% of all cases in high-income countries (Dhana, 2013; Sloan and Parris, 2014). This is a result of a decrease in their immune system’s ability to clear pathogens, due to the HIV suppression of their CD4^+^ T-cell count (Pappas et al., 2010; Kwon-Chung et al., 2014; Gibson and Johnston, 2015). In an immunocompetent host, a Th-1 type CD4^+^ T-cell response would typically clear the infection relatively quickly, with little to no symptoms (Pappas et al., 2010; Gibson and Johnston, 2015). However, in an HIV or immunocompromised host, their immune systems are unable to remove the fungal pathogens from their lungs, leading to the development of a cryptococcal infection (Pappas et al., 2010; Gibson and Johnston, 2015; Garelnabi and May, 2018). After initial infection, *C. neoformans* can traffic from the lungs to the host’s central nervous system (Garelnabi and May, 2018). This leads to the development of cryptococcal meningitis, which has a 40% fatality rate, even with the use of antifungal treatments with ideal conditions (Rajasingham et al., 2017; Patel et al., 2018). For many individuals with severely compromised immune systems, complete removal of the cryptococcal infection is impossible, resulting in a lifelong commitment to the use of antifungal therapies to keep the infection at bay (Coelho and Casadevall, 2016). Current estimates show in AIDS patients, roughly 152,000 cases of cryptococcal meningitis occur each year, with an average of 112,000 yearly deaths (Rajasingham et al., 2022). To help prevent fatalities from cryptococcal infections, early diagnosis is crucial. However, the use of potent fungicidal drugs in combination with fungistatic drugs are still important for the treatment of the disease. Without them, cryptococcal meningitis is fatal (Chen et al., 2010; Dhana, 2013; Sloan and Parris, 2014; Guo et al., 2016). The World Health Organization (WHO) recommended treatment regimen for cryptococcal meningitis in AIDS patients involves three phases: (a) Induction (2 weeks of treatment), (b) Consolidation (8 weeks of treatment), and (c) Maintenance (6–12 weeks or until HIV is controlled by Highly Active Antiretroviral Therapy (HAART)) (Sloan and Parris, 2014). This treatment requires a combination of antifungal therapies over a course of 6–12 months depending on the drug availability. Unfortunately, reduced availability of the drugs for treatment of cryptococcal meningitis continues to be an issue in most Asian and African countries where the disease is most prevalent (Sloan and Parris, 2014).

As the prevalence of dangerous fungal infections continues to rise, the importance of antifungal drugs has risen significantly (Perlin et al., 2017; Fuentefria et al., 2018). Despite many advances in antifungal therapies over the past few decades, the antifungal treatment options are currently limited to only four structural classes of drugs – polyenes, azoles, 5-fluorocytosine, and echinocandins (Perlin et al., 2017; Van Daele et al., 2019). These classes are divided into their respective group based on their mechanism of action (Perea and Patterson, 2002; Pappas et al., 2010). Each of the above-mentioned classes of antifungal therapies have limitations in relation to effectiveness, toxicity and/or the development of drug resistance (Fuentefria et al., 2018). Moreover, the emergence of intrinsic resistance and the ongoing evolution of drug resistant strains has put weight on the limited selection of antifungals available and contributes to the challenge of treating these infections (Perfect, 2017; Wiederhold, 2017; Geddes-McAlister and Shapiro, 2019; Bermas and Geddes-McAlister, 2020). Therefore, the discovery of novel antifungal therapies is critical for fighting these deadly infections (Perlin et al., 2017).

Melanins are dark, negatively charged, hydrophobic pigments that are naturally produced by a multitude of microbes, including bacteria and fungi (Casadevall et al., 2000; Nosanchuk and Casadevall, 2006; Eisenman and Casadevall, 2012; Garcia-Rubio et al., 2020). Eumelanin is a black-brown variety of melanin synthesized by phenoloxidases within a select number of microbes from 3,4-dihydroxyphenyalanine (DOPA) (Hogan et al., 1996; Nosanchuk and Casadevall, 2006; Eisenman and Casadevall, 2012). This variety of melanin is comprised of two monomers, 5,6-dihydroxyindole (DHI) and 5,6-dihydroxyindole-2-carboxylic acid (DHICA) (Selvaraju et al., 2015). The unique properties of the pigments are suggested to have a potential application in the field of medicine (Eisenman and Casadevall, 2012). Melanin production has been associated with increased virulence for various pathogenic microorganisms due to its ability to reduce host defenses by means of antimicrobial mechanisms, including protection from oxidative stress (Nosanchuk and Casadevall, 2006). The ability to protect microbes from the defenses of the host could be relevant to the development of antimicrobial therapies since the use of antimicrobials in tandem with the host immune defenses can increase the effectiveness of some antimicrobial therapies (Nosanchuk and Casadevall, 2006; Adhikari et al., 2022). As such, melanin could be a potential target for the discovery of future antimicrobial therapies. A recent study by *Adhikari* et al. utilized vanillin for the synthesis of a eumelanin-inspired indolyenepheneethylene synthetic compound, EIPE-1 (Adhikari et al., 2022). Through the application of synthetic approaches for derivatization of the methyl-4,7-dibromo-5,6-dimethoxy-1-methyl-1H-indole-2-carboxylate (DBI), a eumelanin-inspired indole core decorated at the 4- and 7- positions, EIPE-1 was prepared as a potential new antimicrobial agent (Selvaraju et al., 2015; Adhikari et al., 2022; Reed et al., 2023). EIPE-1 has two bactericidal moieties ligated to the DBI core that were intended to exhibit similar antibiotic mechanisms to cationic cell-wall disrupting compounds (Baker et al., 1941; Velkov et al., 2013; Adhikari et al., 2022). This compound demonstrated antimicrobial effects against 13 strains of gram-positive bacteria, including two methicillin resistant strains (Adhikari et al., 2022; Reed et al., 2023). Thus, we hypothesized that EIPE-1 may be effective as an antifungal treatment. In this article, we report the antifungal activity of EIPE-1 and its effects on the medically relevant fungal pathogen, *Cryptococcus neoformans*.

## Materials and methods

### Reagents

Unless otherwise stated, chemical reagents and plasticware were obtained from Fisher Scientific (St. Louis, MO). PBS used in washing of cryptococcal cells was obtained at a 10X concentration and diluted 1:10 with deionized water, then sterilized before use. The medium used in Minimum Inhibitory Concentration (MIC) and Minimum Fungicidal Concentration (MFC) Assays was RPMI 1640 supplemented with 0.165 M morpholinepropanesulfonic acid (MOPS), pH 6.9–7, filter-sterilized using a 0.22 μm filter. The cell culture medium used in cytotoxicity experiments was DMEM supplemented with 10% heat-inactivated fetal bovine serum (FBS), 10% NCTC-109, 1% non-essential amino acids, 100 U penicillin/ml, and 100 μg streptomycin/ml, filter-sterilized using a 0.22 μm filter. All mammalian cell cultures were incubated at 37° C, 5% CO_2_ in humidified environments.

### *Cryptococcus* cultures

*Cryptococcus neoformans* strains H99 (serotype A, mating type α) (gift of John Perfect, Duke University Medical Center, Durham, NC), Cn145a (serotype A), and *Cryptococcus gattii* strains R265 (serotype B), R272 (serotype B), R4247 (serotype C), and WSA87 (serotype C) (gift of Brian Wickes, University of Texas Health Science Center, San Antonio, TX), and *Cryptococcus deneoformans* strain 52D (serotype D) were stored at −80°C in 15% glycerol stocks and were plated on yeast extract peptone-dextrose (YPD) (BD Difco; Franklin Lakes, NJ) agar plates. Prior to experiments, individual cryptococcal strains were incubated with shaking in YPD broth for 18 h at 30°C. Cells were collected through centrifugation and washed three times in sterile phosphate-buffered saline (PBS). The cells were quantified using Trypan blue exclusion in a hemacytometer and were resuspended in required medium at the concentration needed for each experiment.

### Synthesis of EIPE-1

**Figure 1:**
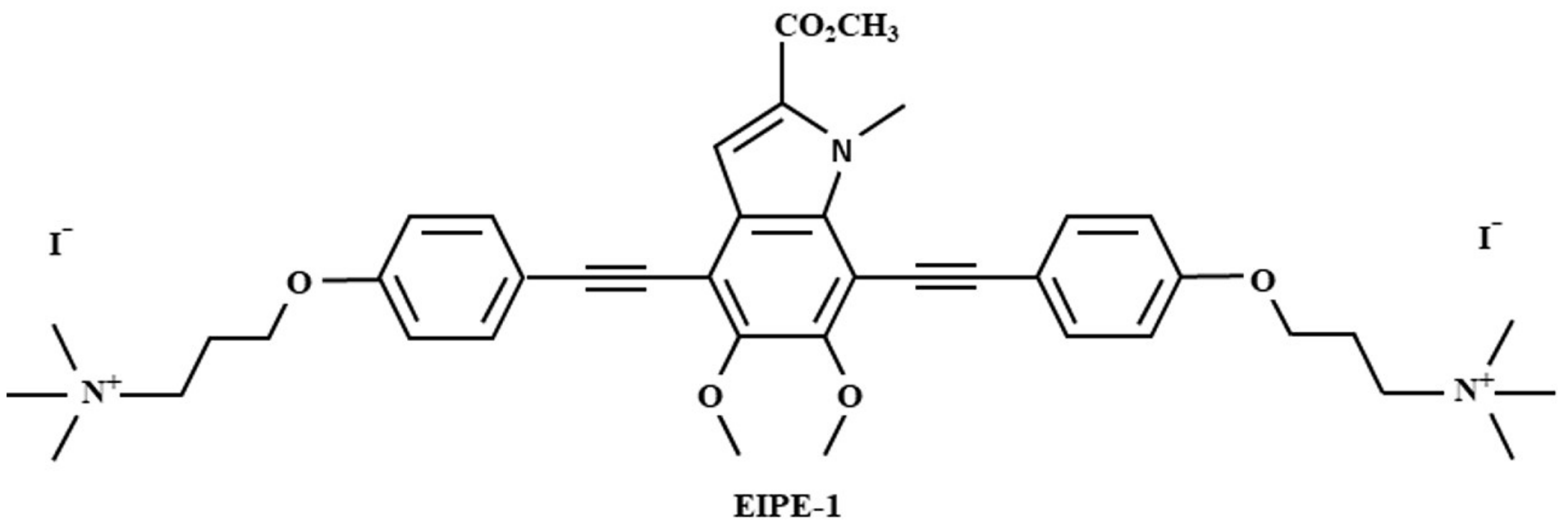
Molecular structure of eumelanin-inspired indolyenepheneethylene (EIPE-1).

3,3′-(((5,6-dimethoxy-2-(methoxyxarbonyl)-1-methyl-*1H-*indole-4,7-diyl)bis(ethyne-2,1-diyl))bis(4,1-phenylene)bis(oxy))bis(*N,N,N*-trimethylpropan-1-aminium) iodide (EIPE-1) (Figure 1) was synthesized and provided by Dr. Nelson’s laboratory (Adhikari et al., 2022; Reed et al., 2023). EIPE-1 powder was then reconstituted with dimethyl sulfoxide (DMSO) to a stock concentration of 5 mg/mL. Dilutions to working concentrations for experiments were made into the media used for each experiment.

### Minimum inhibitory concentration and minimum fungicidal concentration assays of *Cryptococcus*

MIC assays were conducted according to CLSI guidelines (CLSI, 2017 #3098). Briefly, either EIPE-1 or Amphotericin B (AmB) was diluted in RPMI-MOPS, pH 7.0 and evaluated in a two-fold dilution in a concentration range of 100 μg/mL to 0.0488 μg/mL. Dilutions were made in RPMI-MOPS, in a 96-well microtiter plate. A single cryptococcal strain was added to all wells at 0.5×10^3^/ml. Growth controls included the cryptococcal strain grown in media alone. Plates were incubated at 35°C in a humidified incubator for 48 h. The optical densities at 490 nm were measured with a Synergy HTX multi-mode plate reader (BioTek, Winooski, VT), and plates were also visually inspected for turbidity (indicating growth). For MFC assays, dilutions including and above the determined MIC concentration were plated on YPD plates and incubated at 30°C in for 48 h. MFC was defined as the concentration that permitted less than three colony forming units (CFUs), or no growth on the plates, indicating a reduction in 99.9% of the initial inoculum (fungicidal). In other words, the compound has a 99.9% fungicidal activity against the yeast cells (Ernst et al., 1996; Graybill et al., 1997; Espinel-Ingroff et al., 2002; Leite et al., 2014).

### Checkerboard assay using EIPE-1 in combination with AmB against *Cryptococcus neoformans*

Checkerboard assays were conducted using a method previously described, to determine the antifungal activity of EIPE-1 in combination with AmB (Bonifácio et al., 2019; Nelson et al., 2021). EIPE-1 or AmB were evaluated in a two-fold dilution as described in the MIC protocol above. Dilutions were made in RPMI-MOPS, in a 96-well microtiter plate. A single cryptococcal strain was added to all wells at 0.5×10^3^/ml for evaluation of efficacy of combinations. Controls used were EIPE-1 only (row H), AmB only (Column 10), growth control (column 11), and media control (column 12). Plates were incubated at 35°C in a humidified incubator for 48 h. The optical densities at 490 nm were measured with a Synergy HTX multi-mode plate reader (BioTek, Winooski, VT). Results were analyzed using the Fractional Inhibitory Concentration Index (FICI), a non-parametric model based on the Loewe additivity theory to determine the interaction of the combination of EIPE-1 and AmB, where FICI ≤ 0.5 is synergistic, FICI 0.5–4 is indifferent, and FICI ≥ 4 is antagonistic. FICIs were defined as the sum of individual FICs (FICI = FIC_Am B_ + FIC_EIPE-1_), with FICs being defined as the MIC derived from the combination therapy divided by their MIC alone (FIC = MIC_Combination_/MIC_Alone_). Off-scale MICs were considered to be the highest or lowest concentration tested in the microdilution assay (Bonifácio et al., 2019; Nelson et al., 2021).

### Cytotoxicity assay with EIPE-1

To test the cytotoxicity of EIPE-1 on mammalian cells, individual cell lines, including the human cervical epithelial cell line HeLa, murine fibroblast cell line McCoy, and human lung epithelial cell line A549 (all acquired from ATCC), were tested using the CyQUANT™ LDH Cytotoxicity Assay, fluorescence (Invitrogen). For this, cells were grown in cell culture medium according to ATTC guidelines at 37° C with 5% CO_2_. The Cytotoxicity Kit was used according to the manufacturer’s instructions. Briefly, mammalian cells were added in triplicate to wells of a 96-well plate (1 × 10^6^ cells/ml in 100 μL). EIPE-1 was prepared similarly to the MIC assay (1X MIC, 2X MIC, and 10X MIC) except cell culture media was used for dilutions and was added at 10 μL per well. Negative controls included media alone and untreated cells, and the positive control included fully lysed cells. Plates were incubated for 24 h at 37°C, 5% CO_2_. After incubation, 50 μL of reaction mixture was added and incubated for 10 min at room temperature. Following incubation, 50 μL of stop solution was added to each sample. Fluorescence was measured on a Synergy HTX multi-mode plate reader (BioTek) with filters for 560/25 (excitation) and 590/20 (emission). Cytotoxicity of EIPE-1 was conducted in two independent experiments (*n* = 2) with each cell line, with each condition performed in triplicate. Percent cytotoxicity was defined as the fluorescence of experimental wells (cell line and EIPE-1) divided by negative control untreated cells. Greater than 30% cytotoxicity is considered cytotoxic, whereas lower percentages (<30%) were considered non-toxic (ISO10993-5, 2009).

### Electron microscopy

In order to visualize fungal cells using electron microscopy, a higher quantity of fungal cells (10×10^6^ cells) was used. Prior to conducting electron microscopy experiments, we determined the MIC of EIPE-1 using a higher number of cryptococcal cells (strain H99). We followed the same MIC protocol above and determined the MIC was 6.25 μg/mL for this number of cells. *Cryptococcus neoformans* cells were resuspended in RPMI-MOPS, pH 7.0, at a concentration of 10 × 10^6^ cells/ml. For compound treated samples, EIPE-1 was added at 6.25 μg/mL. Negative controls included untreated *C. neoformans* strain H99 cells incubated under the same conditions for each time point. The fungal cells were incubated at 35°C in humidity for 4 h, 6 h, 8 h, or 12 h to detect changes in cell morphology over time. The cells were collected by centrifugation. The pellet was resuspended in 2.0% glutaraldehyde in 0.1 M cacodylate buffer at a volume of 1 mL for a minimum of 2 h and processed for scanning electron microscopy (SEM) or transmission electron microscopy (TEM) at the Oklahoma State University (OSU) Microscopy Laboratory (Stillwater, OK) using their provided protocols.

### Examination of *Cryptococcus* via scanning electron microscopy

Fixed *C. neoformans* cells were collected by centrifugation and transferred to a 12-well plate. Cells were rinsed three times in a buffered wash (30% cacodylate buffer, and 6.15% sucrose) at fixed intervals of 15 min. *C. neoformans* were incubated in osmium tetroxide (1% OsO_4_) for 1 h in a 36-well plate with a clear coverslip. Following incubation, *C. neoformans* was rinsed three times in a buffered wash at fixed intervals of 15 min. *Cryptococcus neoformans* were dehydrated in ethanol (50, 70, 90, 95, and 100%) in increasing percentages three times at fixed intervals of 15 min. *Cryptococcus neoformans* were washed two times with hexamethyldisilane at a time interval of 5 min. Coverslips were removed and placed on a clear sheet for 12 h until dried. Coverslips were mounted on stubs using silver paint. Sample mounts were covered in an Au-Pd coat by the OSU Microscopy Laboratory. Images were examined with a FEI Quanta 600 field-emission gun Environmental Scanning Electron Microscope with a Bruker EDS X-ray microanalysis system and HKL EBSD system. Images were examined at 10000X and 20,000x for SEM. At least 8 fields per condition were examined.

### Examination of *Cryptococcus* via transmission electron microscopy

Fixed *C. neoformans* cells were collected by centrifugation. Media was removed and *C. neoformans* was rinsed 3 times in a buffered wash at fixed intervals of 15 min. Rinsed cells were resuspended in 1% OsO_4_ at room temperature for 1 h. 1% OsO_4_ was removed. *Cryptococcus neoformans* was rinsed 3 times in a buffered wash at fixed intervals of 15 min. *Cryptococcus neoformans* were dehydrated in ethanol (50, 70, 90, 95, and 100%) in increasing concentrations three times at fixed intervals of 15 min. *Cryptococcus neoformans* were washed three times in propylene oxide for fixed intervals of 15 min. Cells were placed in 1:1 propylene oxide and Poly/Bed for 12 h. *Cryptococcus neoformans* cells were embedded (100% embedding medium) and sliced to 80 nm in thickness by the staff of the OSU Microscopy Laboratory. Images were examined with a JEOL JEM-2100 with Bruker EDS at 8000X for TEM. At least 8 fields per condition per time point were examined.

### RNA purification of *Cryptococcus neoformans*

*Cryptococcus neoformans* at a concentration of 10 × 10^6^ cells/ml was incubated with EIPE-1 and RPMI MOPS using the minimum inhibitory concentration from the previously described MIC assays for this number of cells. Cells were incubated at 35°C in a humid incubator for 6 h, which correlated to the time point we observed morphological changes in the fungal cells by SEM and TEM. Untreated cryptococcal cells incubated under the same conditions were used as controls. RNA was purified using AllPrep© Fungal DNA/RNA/Protein kit (Qiagen) and quantified using the Take3 plate on a Synergy HTX multi-mode plate reader (BioTek). RNA was determined pure at a 260/280 ratio of 2.0 (ISO20395, 2019). RNA experiments were conducted in triplicate.

### RNA analysis

RNA was sent for sequencing to Novogene Corp (Sacramento, CA). Fungal RNA-sequencing was conducted using SMARTer Stranded V2 library prep and samples were sequenced on the Illumina Platform (PE150 Q30 ≥ 80%) (Novogene Corp). Gene expression was compared between each untreated *C. neoformans* strain H99 incubated for 6 h compared to H99 treated with EIPE-1 for 6 h. This time point was chosen because the fungal cells were still alive, but initial microsocopy studies showed changes in cell wall/membrane were observed starting at 4 h incubation. Statistics were performed by Novogene, and statistically significant differentially-expressed genes (DEG) in the treated vs. untreated cells were reported. Differentially-regulated genes and their reported functions were examined using FungiDB – Fungal & Oomycete Informatics Resources (Amos et al., 2022).

### *Galleria mellonella* infection

*Galleria mellonella* larvae (Carolina Biological Supply, Burlington, NC) were briefly examined for melanization before storage in groups of ten. Prior to experiments, *G. mellonella* were removed from food for 24 h. Larvae were washed in 70% ethanol and ampicillin (1 mg/mL) or Rifampicin (1 mg/mL). *Galleria mellonella* larvae were given an injection into the last proleg with 10 μL of *C. neoformans* H99 (1×10^4^ cells/ml), heat-killed *C. neoformans* H99 (1×10^4^ cells/ml), or PBS (Mylonakis et al., 2005; Fuchs et al., 2010; Kay et al., 2019). Following a 2 h incubation period at room temperature, the larvae were injected with 10 μL of EIPE-1 at 15 μg/mL, 20 μg/mL, or 25 μg/mLdiluted in PBS (treatment) or with 10 μL PBS (control) in the second to last proleg (Mylonakis et al., 2005; Tsai et al., 2016; Kay et al., 2019). *Galleria mellonella* were incubated at 37°C and were examined every 12 h for 10 days. Every 12 h, survival was checked and cocoons were removed to arrest the *G. mellonella* in their larval stage (Sprynski et al., 2014). *Galleria mellonella* larvae were considered dead following full-body melanism (turning brown/black) and immobility (Kay et al., 2019).

### Statistical analysis

Data analyses were conducted using GraphPad Prism version 5.00 for Windows (GraphPad Software, San Diego, CA). Depending on the data collected and interaction observed between cryptococcal cells and the compound, the one-way ANOVA with the Tukey’s multiple comparison test was used to compare the data. For *G. mellonella* studies, the log-rank test was used to compare survival rates.

## Results

### EIPE-1 inhibits cryptococcal growth

**Figure 2:**
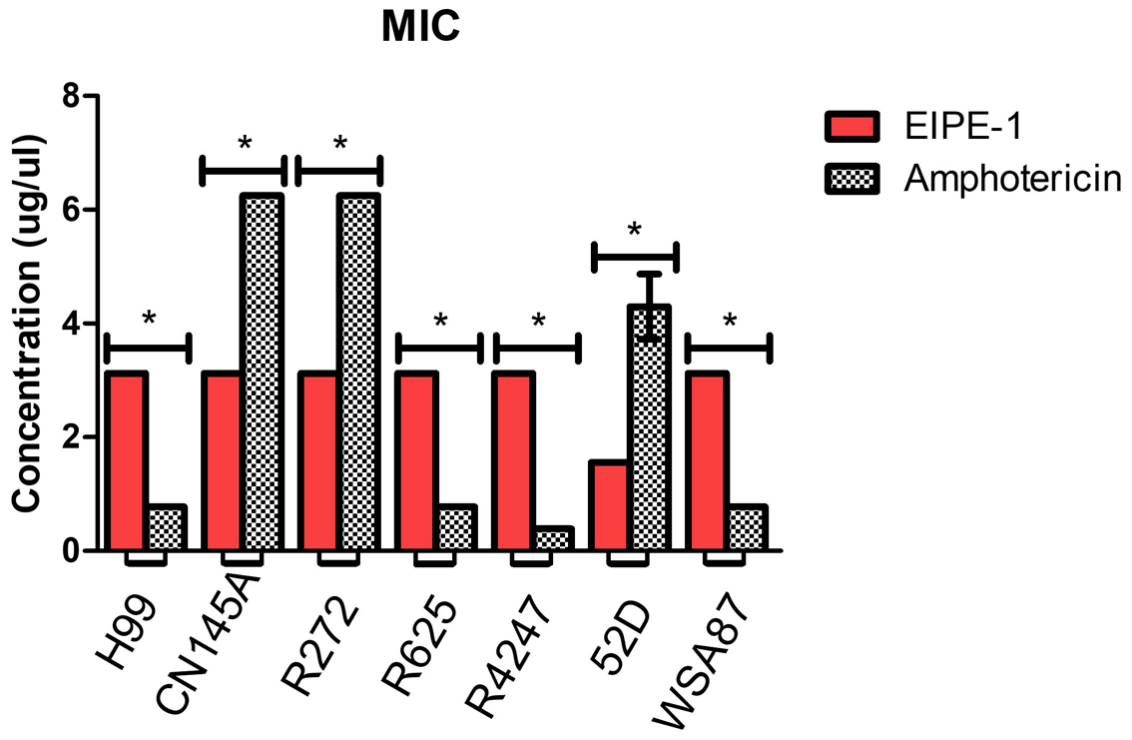
Minimum Inhibition Concentration of EIPE-1 and Amphotericin B against Cryptococcal Strains. *Cryptococcal* yeast cells (strains H99, Cn145a, R265, R272, R4247, WSA87, or 52D) were incubated in RMPI-MOPs alone, in RPMI-MOPS with either EIPE-1, or Amphotericin B in a two-fold dilution for 48 h at 35°C, with humidity. Optical Densities were determined using a multi-mode plate reader. Data shown are from two independent experiments with each cryptococcal strain and means ± SEM are shown. Statistical significance (*p* < 0.05) is shown with an asterisk *. Some strains had no variation between experiments and do not have an error bar.

To determine the antifungal activity of the compound EIPE-1 against cryptococcal strains (H99, Cn145a, R272, R2625, R4247, 52D, and WSA87), we conducted minimum inhibitory concentration (MIC) assays. AmB is an established antifungal drug used against *C. neoformans* in immunocompromised patients, therefore it was used as a control compound for MIC value comparison against EIPE-1 (Perfect et al., 2010; Sloan and Parris, 2014). Statistical analysis showed a significant difference (*p < 0.05*) in antifungal activity following incubation with EIPE-1 compared to *C. neoformans* alone or AmB in RMPI-MOPS (Figure 2). The AmB MIC value had high variation between cryptococcal strains ranging from 0.39 to 6.25 μg/ml. The MIC of EIPE-1 in our assay was 3.125 μg/mL against *C. neoformans* strain H99, *C. gattii* strains R272, R625, and R4247. The MIC value of EIPE-1 was 1.56 μg/mL against *C. neoformans* strain Cn145a, the *C. deneoformans* strain 52D, and the *C. gattii* strainWSA87 (Figure 2). However, despite the variation in the EIPE-1 MIC data, it demonstrates that EIPE-1 can inhibit cryptococcal growth of multiple cryptococcal strains at low concentrations.

To determine whether the antifungal activity demonstrated by EIPE-1 is fungistatic or fungicidal, we conducted minimum fungicidal concentration (MFC) assays. The results of our YPD plates displayed no visible CFUs present after 48 h incubation. This indicates that EIPE-1 is fungicidal at the MIC concentration for each cryptococcal strain.

The *in vitro* interaction of antifungal therapy combinations can have a greater efficacy than the sum of their individual actions, such as seen in the current cryptococcal meningitis treatment guidelines that advises treatment via combination drug therapy (Perfect et al., 2010; Sloan and Parris, 2014; Nelson et al., 2021). Therefore, we tested the synthetic compound EIPE-1 in combination with AmB against *C. neoformans* H99 using a checkerboard assay and categorized the results by the FICI. Both drugs maintained their individual MICs as determined above. Each EIPE-1/AmB combination had an average FICI between 1.17–1.19, placing them in the indifferent category (0.5–4.0). This result is not dependent on the concentration of EIPE-1 used.

### EIPE-1 is non-cytotoxic to mammalian cells

**Figure 3:**
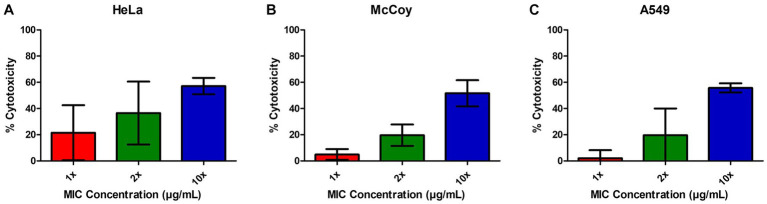
EIPE-1 is not cytotoxic to mammalian cells. **(A)** Human cervical epithelial cell line HeLa, **(B)** Murine fibroblast cell line McCoy, and **(C)** human lung epithelial cell line A549 were incubated in RPMI-MOPS, in RPMI-MOPS with either 6.25 µg/ml EIPE-1, 12.5 µg/ml EIPE-1, or 62.5 µg/ml EIPE-1 for 24 h at 37°C, 5% CO_2_. Cytotoxicity was defined as greater than 30%. Fluorescence was measured on a Synergy HTX multi-mode plate reader. Data displayed are the mean ± SEM of results of 2 independent experiments.

In order to determine the relative cytotoxicity of EIPE-1 to mammalian cells, the CyQUANT™ Cytotoxicity Assay Kit was used with three different mammalian cell lines, including the murine fibroblast cell line McCoy, human lung epithelial cell line A549, and the human cervical epithelial cell line HeLa. EIPE-1 was shown to have a cytotoxicity of <30% (non-toxic) at the MIC concentration tested in all cell lines (Figures 3A–C). At 2X concentration, EIPE-1 was non-toxic for two of the three cell lines, but at 10X concentration, EIPE-1 was toxic (>30%) for all three cell lines. However, since the compound cytotoxicity was less than 30% at the 1X and 2X MIC concentration in most cell lines, the compound was determined to be non-toxic to mammalian cells (ISO10993-5, 2009).

### Electron microscopy reveals structural changes to cryptococcal cells

**Figure 4:**
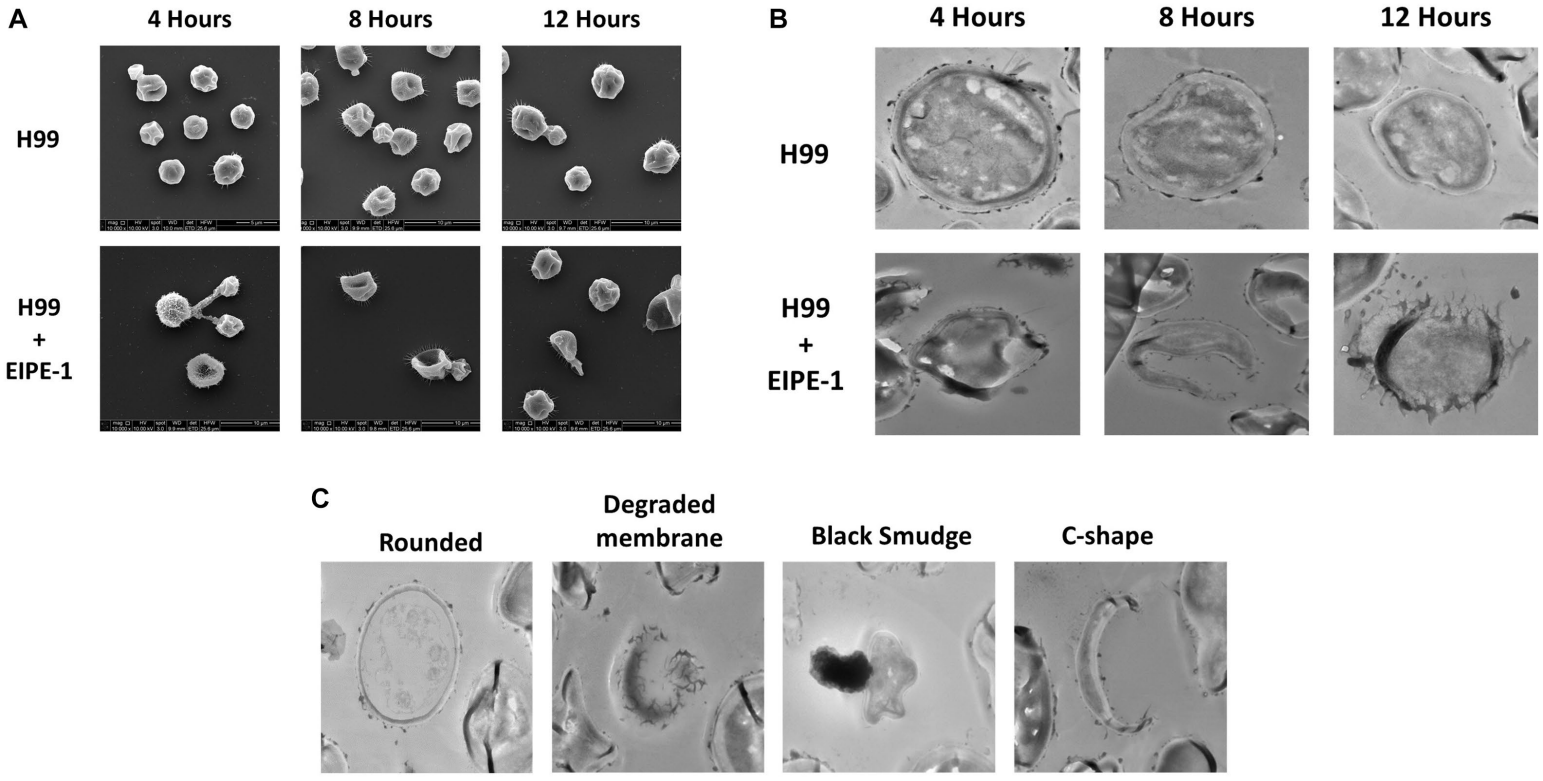
Electron Microscopy of *C. neoformans* with EIPE-1 shows structural changes. *C. neoformans H99* were grown in the presence of EIPE-1 at the calculated MIC of 1.749 μg/mL, for 4, 6, 8, and 12 h, fixed with 2.0% glutaraldehyde in 0.1 M cacodylate buffer, prepared for electron microscopy, and examined for TEM or SEM. **(A)** SEM of *C. neoformans* displayed structural changes (as indicated by the arrows) to the cells incubated with EIPE-1 for 4, 8, and 12 h, but not in the untreated cells. **(B)** TEM of *C. neoformans* displayed structural changes (arrows) to cells treated with EIPE-1 for 4, 8 and 12 h, but not in the untreated cells. **(C)** TEM of *C. neoformans* cells treated with EIPE-1 for 12 h cells displayed four variations in structure – rounded cell (undamaged), degraded membrane, black smudge present over the cell, and c-shape. Magnification is 10,000X for SEM and 8,000X for TEM. Images are representative of at least 8 fields per condition per time point examined.

To understand the mechanism of action of EIPE-1, SEM and TEM analyses were conducted. SEM and TEM can provide vital information about the surface and internal structures of cells (Nixon, 1964; Koga et al., 2021). *Cryptococcus neoformans* strain H99 cells were incubated with EIPE-1 at 4 h, 8 h, or 12 h, following which cells were prepared for electron microscopy. SEM images displayed cell wall/membrane damage as early as 4 h post-incubation (Figure 4A). Damage was indicated by c-shaped cells, mis-shaped cells, etc. (see arrows Figure 4A), indicative of dead/dying cryptococcal cells (Hole et al., 2012). To determine further the effects of EIPE-1 on the cell wall and membrane of the cryptococcal cells, TEM was conducted. TEM allows the internal structures of the cell to be imaged by sectioning of the sample (Winey et al., 2014). *Cryptococcus neoformans* cells were incubated with EIPE-1 at 4 h, 8 h, or 12 h. Time points remained the same as with the SEM to provide a comparison between the SEM and TEM images. The TEM images confirmed that the compound is affecting the cell wall and cell membrane of the fungal cells. Four morphologies were identified within the images (Figure 4C). We observed damage to the cell wall and membrane at 8 h and 12 h (Figure 4B). In addition, it appears that the membrane damage results in a leakage of internal contents into the surrounding media (Figures 4B,C). The TEM results confirmed that the compound is affecting these specific cellular structures on the fungal cells. Additionally, TEM showed two other cell morphologies that represented dying or dead cells, including c-shaped cells. The images also include cells with degraded membranes and a black smudge. TEM incorporates the use of heavy metals to prevent electrons from passing through the prepared sample. These metals bind to regions concentrated with DNA and proteins, or components of the cell that are rich in lipids. In a bright-field TEM, these regions, and regions high in mass density tend to appear dark in the imaging to allow contrast. Thus, the black region (smudge) observed in the TEM images could be a representation of regions rich in DNA, protein or lipids (Dempster, 1960; Belazi et al., 2009; Klein et al., 2015; Lange et al., 2021). Nucleic acid or DNA, protein and lipids make up a majority of the internal macromolecules of a living eukaryotic cell. Due to the composition of the internal cellular components of *C. neoformans*, it is likely the black region observed in the TEM images was comprised of internal structures leaking into the media from a pore present in the cellular wall or membrane of the fungal cell (Schie et al., 2016). As assessed by both the SEM and TEM images, the compound appears to affect the cell wall and membrane of the *C. neoformans* cells, leading to cell lysis.

### EIPE-1 treatment affects cryptococcal gene expression

To understand the effect of EIPE-1 on fungal gene expression, we were interested in identifying differential gene expression between *C. neoformans* incubated with EIPE-1 compared to control. Purified RNA was sent for sequencing at Novogene (Novogene Corp, Sacramento, CA). The analyses identified 4,936 statistically significantly differentially expressed genes (DEG) between untreated and EIPE-1 treated cryptococcal cells. Of these genes, 2,486 were significantly upregulated and 2,450 genes were significantly down-regulated. Due to a limitation on the information available for *C. neoformans* strain H99, one third of the greatest DEGs listed (Table 1) have unknown functions. However, our analyses of FungiDB showed that the genes with available information have roles in metabolic processes, stress response, and virulence of the cells (Amos et al., 2022). Descriptions of the top thirty differentially-regulated genes and their putative functions are shown (Table 2). Furthermore, analyses identified 91 enriched pathways through the KEGG online database. Of these 91 pathways, none were statistically significant. The top five active pathways and their *p*-values are shown (Table 2). These pathways are involved in amino acid biosynthesis, carbon metabolism, ribosome formation, and the replication of DNA (Amos et al., 2022). We further filtered through the list of genes and identified several DEGs involved in *C. neoformans* viability, capsule biosynthesis, capsule attachment and remodeling and ergosterol biosynthesis (Table 3).

**Table 1:** Gene descriptions of the top 30 differentially expressed genes.

Gene ID	Gene name	Log2foldChange	*p*-adjusted	Gene description
CNAG_01683	STL1	−2.362044974	0*	Sugar transporter
CNAG_05387	*–*	1.891607373	0*	MFS glucose transporter mfs1
CNAG_01577	*–*	−2.794167594	0*	NADP-specific glutamate dehydrogenase
CNAG_06150	*–*	2.515378205	0*	Heat shock protein 90–2
CNAG_04630	YAP2	1.495267376	0*	Hypothetical protein
CNAG_01750	*–*	1.7658259	0*	Heat shock protein 70 kDa protein
CNAG_03347	*–*	2.398469689	0*	Heat shock protein 78, mitochondrial
CNAG_02701	*–*	3.704685826	0*	Hypothetical protein (from the BAG domain)
CNAG_00799	*–*	−3.488416798	0*	Cellulose; glucan 1, 3-beta-glucosidase A (putative)
CNAG_04857	*–*	−3.481049556	0*	Hypothetical protein
CNAG_07493	*–*	3.659539832	0*	Hypothetical protein
CNAG_01277	*–*	−2.997262312	0*	Hypothetical protein
CNAG_04891	*–*	2.882029514	0*	Hypothetical protein (from the Ricin-type beta-trefoil lectin domain)
CNAG_01138	CCP1	−2.54156321	0*	Cytochrome c peroxidase, mitochondrial
CNAG_07492	*–*	3.896665753	0*	Hypothetical protein
CNAG_04862	*–*	−3.718177427	0*	Glutamate synthase (NADPH) (putative)
CNAG_00023	*–*	2.919372523	0*	Hypotheical protein
CNAG_02691	*–*	2.940607543	0*	Hypothetical protein (from the BAD domain)
CNAG_03891	*–*	2.148070873	7.67E-302	Heat shock protein 60, mitochondrial
CNAG_00100	*–*	1.982554272	1.16E-293	Heat shock protein sti1 homolog
CNAG_03162	*–*	1.696322184	1.47E-292	BH3 domain-containing protein bxi1; Bax inhibitor 1
CNAG_06244	*–*	1.988950832	8.28E-292	Hypothetical protein
CNAG_03385	PCL103	−2.953559223	3.29E-287	G1/S-specific cyclin PCL1
CNAG_06963	*–*	−3.077006018	7.14E-276	Hexose transporter HXT10
CNAG_06623	*–*	−1.979003167	9.43E-274	Myo-insitol (putative)
CNAG_05741	*–*	2.013204228	3.10E-264	Hypothetical protein (from the Thioesterase-like superfamily)
CNAG_04183	*–*	−2.558639688	5.75E-257	Hypothetical protein
CNAG_03198	*–*	−1.552579938	2.75E-254	40S ribosomal protein
CNAG_07347	*–*	1.502356026	2.96E-253	Heat shock protein 104

Top 30 most differentially expressed genes in order of *p*-value greatest to least. General gene descriptions were obtained from Novogene RNA analysis. Adjusted *p*-values followed by * are below the lowest reliable number from *R* (*p* > 2.2 × 10^−308^).

**Table 2:** *Cryptococcus neoformans* treated with EIPE-1 show activation of specific pathways.

Pathway names	*p*-value	Total gene count
2-Oxocarboxylic acid metabolism	1.26 × 10^−02^	29
Biosynthesis of amino acids	1.76 × 10^−02^	86
Carbon metabolism	1.93 × 10^−02^	82
DNA replication	2.58 × 10^−02^	30
Ribosome	2.95 × 10^−02^	89

Top 5 activated pathways in order of the *p*-value greatest to least.

**Table 3:** Differently regulated genes associated with *C. neoformans* capsule and cellular wall biosynthesis.

CNAG ID	Gene annotation	Log2foldChange	*p*-adjusted	Gene description
*Capsule biosynthesis*				
*Down regulated*				
CNAG_00701	*CAS31*	−2.855001654	1.38E-190	Protein involved in gxm O-acetylation
CNAG_04969	*UGD1*	−1.370492283	3.62E-149	UDP-glucose 6-dehydrogenase
CNAG_03322	*UXS1*	−1.529051795	1.20E-99	UDP-glucoronate decarboxylase
CNAG_03735	*CAP4*	−1.706728123	6.73E-60	Beta-1,2-xylosyltransferase
CNAG_07554	*CAP10*	−1.113797564	3.95E-59	Capsular associated protein
CNAG_02885	*CAP64*	−1.009431948	7.19E-52	Capsular associated protein
CNAG_06813	*CAP1alpha*	−0.878994024	8.49E-34	O-glucosyltransferase (putative)
CNAG_02797	*CPL1*	−0.748852674	6.45E-33	Putative secreted protein
CNAG_00697	*UGE1*	−0.750247372	2.14E-31	UDP-glucose 4-epimerase
CNAG_01172	*PBX1*	−1.424192864	1.04E-30	Parallel beta-helix repeat protein
CNAG_00746	*CAS35*	−0.65732178	7.69E-30	Capsular associated protein
CANG_03644	*CAS3*	−1.007364787	4.82E-26	Capsule related protein
CNAG_01283	*CAP5*	−0.701627895	6.94E-19	Beta-1,2-xylosyltransferase
CNAG_05562	*PBX2*	−0.807311393	1.46E-15	Parallel beta-helix repeat protein
CNAG_04312	*MAN1*	−0.446524344	9.43E-13	Mannose-6-phosphate isomerase
CNAG_07937	*CAS1*	−0.49029209	5.20E-12	O-acetyltransferase
CNAG_02036	*CAS4*	−2.019373373	3.23E-10	Putative sugar transporter
CNAG_00600	*Cap60*	−0.382008652	1.31E-08	Capsular associated protein
CNAG_00996	*PMT4*	−0.279274452	9.81E-07	Dolichy-phosphate-mannose-protein mannosyltransferase
CNAG_02581	*CAS33*	−0.317517775	1.47E-06	Capsular associated protein
CANG_00721	*CAP59*	−0.301829932	4.81E-06	Alpha-1,3-mannosyltransferase
CNAG_03096	*UGE1*	−0.301895617	9.49E-05	UDP-glucose 4-epimerase
CNAG_00744	*OCH1*	−0.183517671	4.21E-03	Alpha 1,6-mannosyltransferase
CNAG_00124	*CAS32*	−0.279541152	1.72E-02	Capsule structure designer protein
CNAG_03695	*CAS41*	−0.454412496	2.15E-02	Capsule biosynthetic protein
CNAG_04320	*CPS1*	−0.118036937	5.92E-02	Polysaccharide synthase Cps1p
*Up regulated*				
CNAG_05139	*UGT1*	0.541924929	3.89E-22	UDP-galactose transporter
CNAG_01156	*CAP2*	0.494410329	4.70E-10	Capsular related protein
CNAG_06016	*CAP6*	0.340720348	2.18E-08	Alpha-1,3-mannosyltransferase
CNAG_00926	*-*	0.341139204	1.25E-07	Alpha-1,3/alpha-1,6-mannosyltransferase
CNAG_05148	*CXT1*	0.245910733	1.22E-06	Beta-1,2-xylosyltransferase 1
*Capsule attachment, cell wall attachment, and remodeling*				
*Down regulated*				
CNAG_04245	*CHI22*	−2.717172168	8.04E-252	Chitinase
CNAG_02850	*AGN1*	−3.054787283	1.37E-176	Glucan endo-1,3-alpha-glucosidase
CNAG_02351	*CHI4*	−1.368795394	4.70E-74	Endochitinase
CNAG_06487	*CHS6*	−3.704233375	1.32E-31	Chitin synthase
CNAG_04187	*GWT1*	−1.079701801	8.51E-30	GPI-anchored wall transfer protein 1
CNAG_02860	*EBG1*	−0.416393502	2.18E-19	Endo-1,3(4)-beta-glucanase
CNAG_03855	*–*	−0.876359081	5.06E-11	Phosphatidylinositol glycan, class M
CNAG_06031	*KRE63*	−0.67649535	1.13E-10	Beta-glucan synthesis-associated protein
CNAG_02225	*EXG104*	−0.624871051	3.85E-10	Glucan 1,3-beta-glucosidase
CNAG_02598	*CHI21*	−0.883751652	5.85E-09	Chitinase
CNAG_05574	*–*	−0.404608237	3.51E-05	Phosphatidylinositol glycan, class C
CNAG_00401	*–*	−0.401425384	5.64E-04	Phosphatidylinositol glycan, class U
CNAG_05617	*GPI13*	−0.339455578	1.90E-04	Phosphatidylinositol glycan, class O
CNAG_03442	*–*	−0.462926782	6.023E-03	Phosphatidylinositol glycan, class T
CNAG_05413	*–*	−0.41014114	3.80E-03	Phosphatidylinositol glycan, class Q
CNAG_02283	*–*	−0.530571953	1.99E-02	Glucoamylase
CNAG_01239	*CDA3*	−0.14025053	4.11E-02	Chitin deacetylase
*Up regulated*				
CNAG_06336	*–*	0.708376857	1.98E-52	Glucan 1,3-beta-glucosidase
CNAG_05663	*SCW1*	0.58325895	2.87E-33	Cell wall integrity protein
CNAG_05581	*CHS3*	0.449128753	2.43E-16	Chitin synthase
CNAG_06508	*FKS1*	0.403758545	2.07E-15	1,3-beta-glucan synthase component
CNAG_05818	*CHS5*	0.445328128	5.00E-13	Chitin synthase
CNAG_00546	*CHS4*	0.410978366	4.08E-12	Chitin synthase
CNAG_01230	*MP98*	0.298088628	3.56E-11	Chitin deacetylase 2
CNAG_06659	*HEX1*	0.324315235	8.34E-07	Beta-hexosaminidase
CNAG_03412	*CHI2*	0.293790115	2.95E-06	Chitinase
CNAG_06832	*KRE62*	0.535595208	4.25E-05	Glucosidase
CNAG_03026	*–*	0.302152734	1.62E-05	N-acetylglucosaminylphosphatidylinositol deacetylase
CNAG_01941	*–*	0.298519571	6.24E-04	Glucan synthesis regulatory protein
CNAG_01386	*–*	0.372375349	5.99E-04	Phosphatidylinositol glycan, class P
CNAG_00897	*SKN1*	0.203870908	2.51E-04	Putative glucosidase
CNAG_05673	*–*	−0.238968958	2.28E-04	GPI inositol-deacylase
CNAG_04525	*–*	0.280053426	3.00E-03	Glycosylphosphatidylinositol transamidase
CNAG_06835	*KRE61*	0.159632141	1.69E-03	Glucosidase
CNAG_07636	*CSR2*	0.16676414	2.88E-02	Protoplast regeneration and killer toxin resistance protein
*Ergosterol biosynthesis*				
*Down regulated*				
CNAG_00040	*ERG11*	−1.500820267	2.71E-126	Endochitinase
CNAG_00519	*ERG3*	−1.115800276	6.05E-50	Chitin synthase
CNAG_02896	*ERG130*	−1.284385062	1.04E-36	GPI-anchored wall transfer protein 1
CNAG_03819	*ERG6*	−1.401562387	3.94E-36	Endo-1,3(4)-beta-glucanase
CNAG_00854	*ERG2*	−1.207132683	1.33E-36	Phosphatidylinositol glycan, class M
CNAG_06644	*ERG5*	−0.570266293	6.12E-32	Beta-glucan synthesis-associated protein
CNAG_02830	*ERG4*	−1.009861876	1.04E-28	Glucan 1,3-beta-glucosidase
CNAG_02084	*ERG20*	−0.822662681	6.97E-22	Chitinase
CNAG_00117	*ERG24*	−0.617813827	8.56E-18	Glucan endo-1,3-alpha-glucosidase
CNAG_06534	*HMG1*	−0.163966428	2.38E-02	Endochitinase
*Up regulated*				
CNAG_01737	*ERG25*	0.597438009	3.69E-26	GPI-anchored wall transfer protein 1
CNAG_06001	*ERG8*	0.911533759	9.20E-21	Endo-1,3(4)-beta-glucanase
CNAG_02918	*ERG10*	0.26658416	9.82E-07	Phosphatidylinositol glycan, class M
CNAG_03311	*ERG13*	0.161426965	4.42E-03	Beta-glucan synthesis-associated protein

Genes are in order of *p*-value greatest to least. General gene descriptions were obtained from Novogene RNA analysis and FungiDB: Fungal & Oomycete Informatics Resources.

### EIPE-1 does not clear cryptococcal infection in *Galleria mellonella*

**Figure 5:**
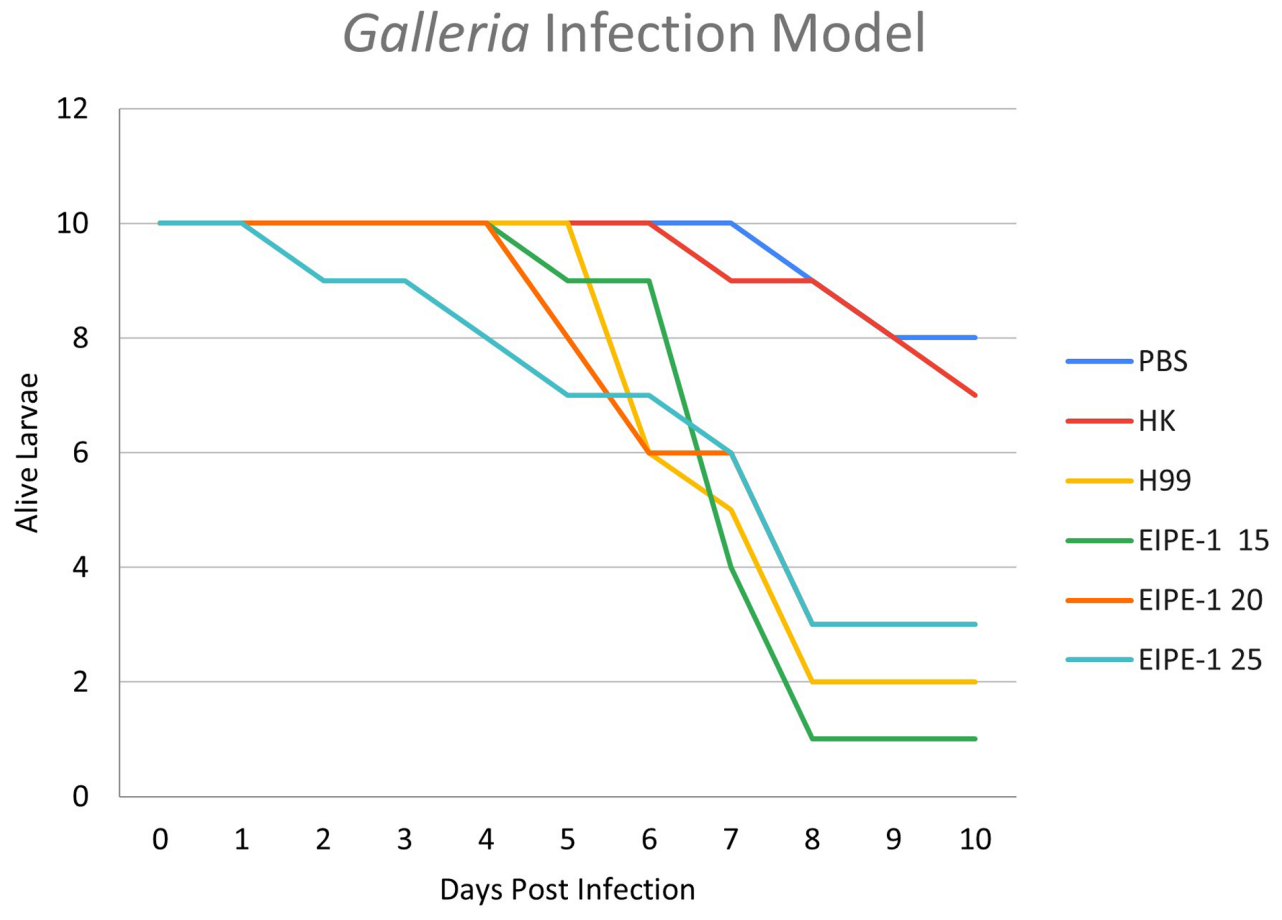
EIPE-1 does not provide antifungal protection during *in-vivo* infection of *Galleria mellonella*. *G. mellonella* larvae were inoculated with either PBS alone or EIPE-1 at the concentration of 15 μg/mL, 20 μg/mL, 25 μg/mL, 50 μg/mL, 100 μg/mL, 150 μg/mL, or 200 μg/mL diluted in PBS. Infected *G. mellonella* were incubated at 37°C and were examined every 12 h for mortality for 10 days. Larvae were considered dead following full-body melanism and immobility. Data are representative of three independent experiments.

To determine the efficacy of EIPE-1 in a living infection model, *G. mellonella* larvae were infected with *C. neoformans* H99 and treated with various concentrations of EIPE-1 as mentioned in the methods. As shown in Figure 5, larvae of the *G. mellonella* inoculated with *C. neoformans* H99 experienced rapid death by day five of infection. Additionally, larvae inoculated with *C. neoformans* and treated with EIPE-1 experienced death at similar time points to H99 alone, regardless of the concentration of EIPE-1 (Figure 5).

## Discussion

Despite advances in antifungal therapies over the decades, antifungals are limited to only 14 individual agents (in 4 classes) that have been approved by the U.S. Federal Drug and Food Administration (FDA) for use in the treatment of fungal infections (Dismukes, 2000; Nett and Andes, 2016). The goal of this study is to explore the potential of EIPE-1 as an effective and non-toxic antifungal for the purpose of increasing the current therapies on the market for the treatment of fungal infections, in particular the infection caused by *C. neoformans* (Perlin et al., 2017; Van Daele et al., 2019). While its original purpose was as a building block for organic semiconductors, it was later discovered that this core has the interesting capability of serving as a foundation for the integration of antibactericidal moieties and possesses intrinsic antimicrobial activity that causes cytoplasmic membrane disruption in gram positive bacteria. Additionally, it was revealed that thirteen different strains from eight gram-positive bacteria, including two methicillin resistant strains were found to be susceptible to EIPE-1 (Adhikari et al., 2022; Reed et al., 2023). While not previously studied in fungal organisms, we found enough evidence to support studying this compound as an antifungal against *Cryptococcus* isolates. Therefore, we decided to study this compound to determine its use as a novel antifungal drug against *Cryptococcus*.

Cells come into contact with environmental stressors (Cowen and Steinbach, 2008). A microbe’s ability to adapt to these stresses present in its surrounding environment is crucial for survival in their biological niches. One key attribute of *Cryptococcus* is its ability to survive in harsh environments via sensing, responding, and adapting to changes for its survival and proliferation. During antimicrobial treatments, the fungal organism senses and initiates stress signal pathways which allows them to adapt (Dismukes, 2000; Cowen and Steinbach, 2008; Fuchs and Mylonakis, 2009; Nett and Andes, 2016). A stress response can be seen during treatment with EIPE-1, as several stress-associated genes recognized in previous literature have been identified in the RNAseq analysis. These genes include but are not limited to the Ricin Beta Lectin superfamily, ATPases associated with diverse cellular activities (AAA+) superfamily, Bcl-2-associated athanogen (BAG)-family proteins and SLC2A (GLUT) family (Doong et al., 2002; Ishikawa et al., 2011; Mueckler and Thorens, 2013; Gallegos et al., 2014; Khan et al., 2022). In addition, we saw a reduction in ribosomal protein translation in our treated populations. Regulation of translation is crucial for *C. neoformans* to adapt to the environmental stressors (Knowles et al., 2021).

This capability of *C. neoformans* to adapt to stressors demonstrates not only its cellular mechanisms, but also its plasticity of its cell wall, which plays a key role in the defense of the cell from environmental stress and maintains integrity of the cell (Rodrigues et al., 2008; Garcia-Rubio et al., 2020; Upadhya et al., 2023). Disruption of the fungal cellular wall by interfering with glucosidases and chitinase may be an important mechanism by which EIPE-1 exerts its antifungal effects. Specifically, during fungal growth, chitinase is involved in the breakdown of chitin and chitosan by hydrolyzing polymers of chitin at the beta-(1-4) linkages. Chitin and chitosan are vital components of the fungal cell wall and have been shown to contribute to the general stability of the cellular wall (Banks et al., 2005; Baker et al., 2009).

All this is an expected response of the cell when exposed to stress and/or apoptotic stimuli of an agent with antifungal capabilities. However, while we believe these genes and transcriptional pathways are the most important for the morphological changes and cellular death observed in the EIPE-1 treated population, we must note that there may be important genes involved that were not identified during initial analyses. Additionally, whereas the *C. neoformans* genome for strain H99 has been previously sequenced, not all the genes have been annotated to determine the function. Moreover, many of the annotated genes of fungal species are generated by comparison of genomes and by automatic sequence analysis pipelines. Therefore, it is possible that important genes were excluded from our analyses, due to these limitations (Janbon et al., 2014). In the future, follow-up studies need to be done to validate the gene expression data.

**Figure 6:**
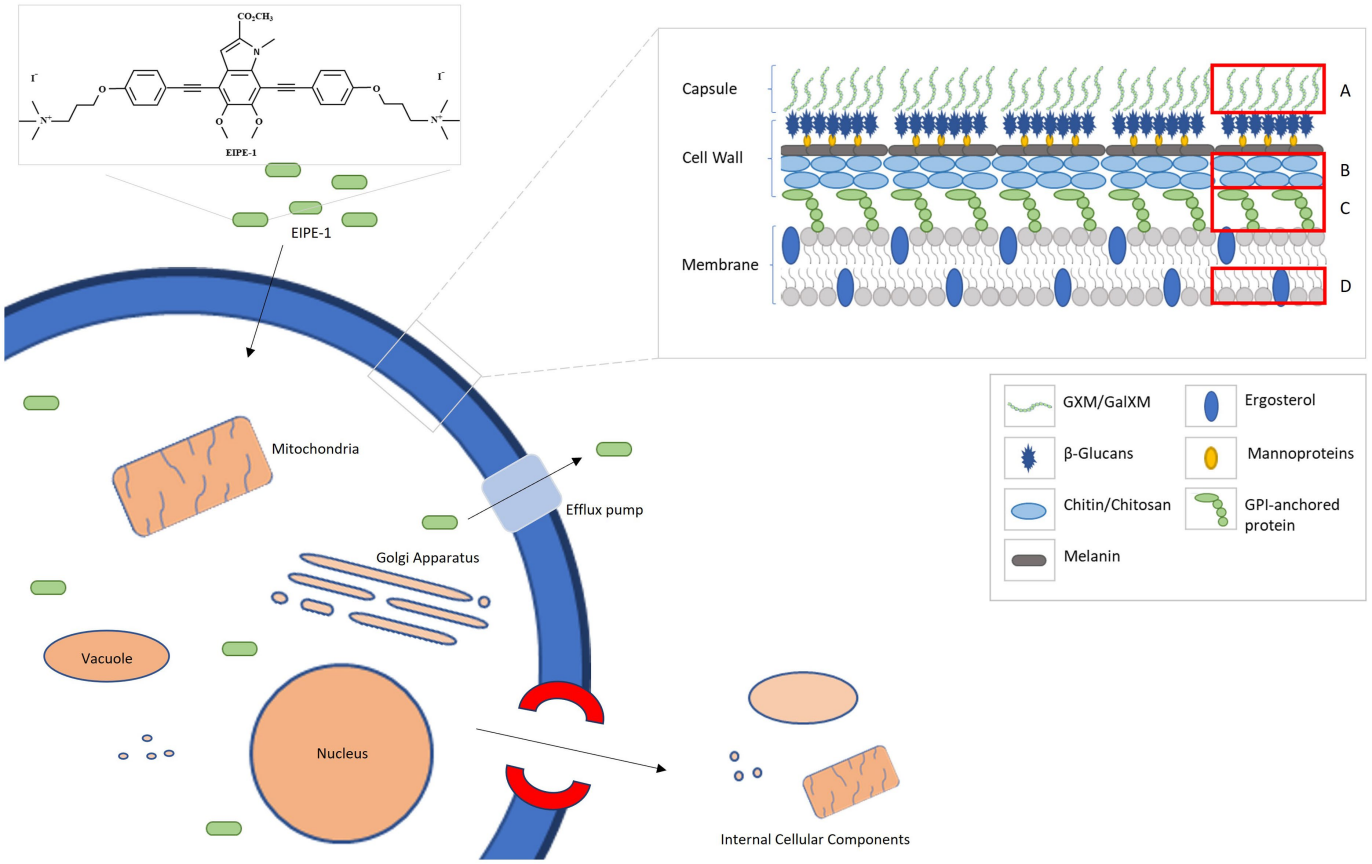
A putative model for the mechanism of antifungal activity of EIPE-1. *C. neoformans H99* have a complex cell wall comprised of chitin, chitosan, a-1,3 glucan, B-1,3 glucan, B-1,6 glucan, mannoproteins and GPI-anchored proteins. Additionally, they possess a capsule that is consistently maintained on the outer cell wall and composed of GXM and GalXM. EIPE-1, a hydrophobic eumelanin inspired molecule, may enter the cell through a passive method and alters components of the cell by a downregulation in genes involved with capsule biosynthesis, cell wall attachment and remodeling, and ergosterol biosynthesis. **(A)** Glucuronoxylomannan (GXM) and Galactoxylomannan (GalXM). **(B)** Chitin and Chitosan. **(C)** GPI-anchored proteins. **(D)** Ergosterol.

Combining all the data from the RNA sequencing and the electron microscopy, we composed a putative model based on the effects of EIPE-1 against *C. neoformans* as displayed in Figure 6. As found in all living eukaryotic cells, beneath the fungal cell wall, *C. neoformans* possesses a plasma membrane that consists of a phospholipid bilayer (Rodrigues et al., 2008; Agustinho et al., 2018; Upadhya et al., 2023; Zhukov and Popov, 2023). This membrane maintains the viability of a cell and prevents the free exchange of molecules from the cytoplasm to the cell’s environment and vice versa (Upadhya et al., 2023). Previously, microorganism membrane permeability to hydrophobic molecules was identified as being pertinent for susceptibility to the molecule (Reed et al., 2023). Since *C. neoformans* has a high cellular surface hydrophobicity due to the presence of mannoproteins, lipids, glucan, and chitin molecules, it could allow the hydrophobic EIPE-1 molecule to passively diffuse across the membrane into the cell (van der Rest et al., 1995; Danchik and Casadevall, 2020; Vij et al., 2020). Once within the cell, it interacts with the cell’s ability to synthesize the cell wall and the membrane. During this process, the cell responds to the presence of EIPE-1 by the up-regulation of genes involved in stress response, including efflux pumps, heat shock proteins, etc. (Doong et al., 2002; Cowen and Steinbach, 2008; Ishikawa et al., 2011; Mueckler and Thorens, 2013; Gallegos et al., 2014; Holmes et al., 2016; Kurop et al., 2021; Khan et al., 2022). Specifically, efflux pumps allow the organism to regulate its internal environment by the removal of antimicrobial substances (Holmes et al., 2016). While the overall function of EIPE-1 is still relatively unknown, it has been shown to interfere with several pathways involved in the biosynthesis of ergosterol, GPI-anchored proteins, GXM/GalXM, chitin, and chitosan. The result of this interference, the cell’s membrane and wall are damaged leading to the formation of a pore and/or breakdown of the cellular membrane. When this occurs, the cell is no longer able to maintain the internal environment and may allow leakage of cellular organelles, as seen in Figure 4C, leading to cellular death.

This model provides perhaps the clearest illustration of the dynamics of EIPE-1 against *C. neoformans*. However, we must recognize that the true mechanism of the synthetic molecule’s antifungal activity against *Cryptococcus* is still largely unknown. It is possible that the damage we are observing in the treated populations could be a downstream effect of the true target of EIPE-1. Future investigations involving the use of *C. neoformans* mutant libraries to pinpoint the molecular target of EIPE-1 are currently underway in our laboratory. While these data suggest that EIPE-1 may have potential as a novel antifungal against *C. neoformans*, we are aware that we do not know the true efficiency of the synthetic compound within an *in vivo* model. There are various factors that can impact the efficiency of a therapeutic treatment in a living model over time from host-pathogen interactions to the distribution, metabolism, and elimination of EIPE-1 from the host’s body. All these can impact the bioavailability and efficiency of the administered drug since less of our drug may remain active or as potent at the target sites of infection (Gillette, 1971; Ekins et al., 2000; Adepu and Ramakrishna, 2021). Additionally, during pathogenesis, *C. neoformans* cells typically interfere with immune cell recognition and phagocytosis with its virulence factors, including melanin production and a capsule composed of galactoxylomannan (GalXM) and glucuronoxylomannan (GXM). These factors add protective features to the fungal cells. For example, the components of the capsule have an anti-phagocytic influence on immune phagocytes, allowing the pathogen to evade phagocytosis (Kozel and Gotschlich, 1982; Kozel et al., 1988; Yauch et al., 2006; Zaragoza et al., 2009; Vecchiarelli et al., 2013; Conn and Wozniak, 2023). Interestingly, our EIPE-1 treated *C. neoformans* cells have a reduction in genes that regulate capsule biosynthesis. While this pathway is not typically required for viability of the yeast since acapsular mutants can survive and replicate *in vitro* (Grijpstra et al., 2009; Tefsen et al., 2014), it is required for virulence *in vivo* (Chang and Kwon-Chung, 1994; Chang et al., 1996), and the absence of capsule results in a reduction in virulence. This indicates that during treatment our *C. neoformans* may remain in a less virulent state, which may also aid our immune cells during pathogen clearance when the correct bioavailability and potency is maintained (Gillette, 1971; Ekins et al., 2000; Adepu and Ramakrishna, 2021). It is important to learn the role our innate immune cells will play during pathogen clearance of *C. neoformans* during EIPE-1 treatment. Studies are ongoing in our lab to understand how treatment with EIPE-1 may affect immune-mediated clearance of *C. neoformans*.

Finally, *C. neoformans* is capable of producing its own melanin in the host in the presence of L-DOPA. This plays an important role in protecting *C. neoformans* from host induced damage due to reactive oxygen species. Melanin is also capable of binding and impacting the effect of antifungal treatments on the fungal cells (Wang and Casadevall, 1994; Zaragoza et al., 2009; Lee et al., 2019). This leads to the question of whether EIPE-1 will be effective against cryptococcal cells when in a melanized form. As previously mentioned, the structure of the EIPE-1 indole core is inspired by eumelanin molecular structure (Adhikari et al., 2022). We do not know if structural similarities will contribute to stronger binding or decrease the susceptibility of *C. neoformans* to the novel compound. Future studies are being conducted on the potential of EIPE-1 on melanized *C. neoformans*.

## Data availability statement

The transcriptome datasets that support the findings of this article are available to the public in the NCBI BioSample database (ncbi.nlm.nih.gov/biosample/) under the accession number PRJNA1052015, samples SAMN38810541, SAMN38810542, SAMN38810543, SAMN38810544, SAMN38810545, and SAMN38810546. Further inquiries can be directed to the corresponding author.

## Ethics statement

Ethical approval was not required for the studies on humans in accordance with the local legislation and institutional requirements because only commercially available established cell lines were used. Ethical approval was not required for the studies on animals in accordance with the local legislation and institutional requirements because only commercially available established cell lines were used.

## Author contributions

BC: Conceptualization, Data curation, Formal analysis, Investigation, Methodology, Writing – original draft, Writing – review & editing. JL: Data curation, Formal analysis, Investigation, Methodology, Writing – review & editing. PC: Data curation, Formal analysis, Investigation, Writing – review & editing. KC: Data curation, Formal analysis, Investigation, Methodology, Writing – review & editing. MaE: Writing – review & editing, Investigation, Methodology. MoE: Investigation, Methodology, Writing – review & editing. TN: Conceptualization, Formal analysis, Funding acquisition, Methodology, Writing – review & editing. KW: Conceptualization, Data curation, Project administration, Resources, Supervision, Writing – review & editing.
